# Slow evolution of sex‐biased genes in the reproductive tissue of the dioecious plant *Salix viminalis*

**DOI:** 10.1111/mec.14466

**Published:** 2018-01-22

**Authors:** Iulia Darolti, Alison E. Wright, Pascal Pucholt, Sofia Berlin, Judith E. Mank

**Affiliations:** ^1^ Department of Genetics, Evolution and Environment University College London London UK; ^2^ Department of Animal and Plant Sciences University of Sheffield Sheffield UK; ^3^ Department of Plant Biology Linnean Centre for Plant Biology Swedish University of Agricultural Sciences Uppsala Sweden; ^4^ Array and Analysis Facility Department of Medical Science Uppsala University Uppsala Sweden; ^5^ Department of Organismal Biology Uppsala University Uppsala Sweden

**Keywords:** codon usage bias, dioecious angiosperms, sex‐biased gene expression, sexual selection

## Abstract

The relative rate of evolution for sex‐biased genes has often been used as a measure of the strength of sex‐specific selection. In contrast to studies in a wide variety of animals, far less is known about the molecular evolution of sex‐biased genes in plants, particularly in dioecious angiosperms. Here, we investigate the gene expression patterns and evolution of sex‐biased genes in the dioecious plant *Salix viminalis*. We observe lower rates of sequence evolution for male‐biased genes expressed in the reproductive tissue compared to unbiased and female‐biased genes. These results could be partially explained by the lower codon usage bias for male‐biased genes leading to elevated rates of synonymous substitutions compared to unbiased genes. However, the stronger haploid selection in the reproductive tissue of plants, together with pollen competition, would also lead to higher levels of purifying selection acting to remove deleterious variation. Future work should focus on the differential evolution of haploid‐ and diploid‐specific genes to understand the selective dynamics acting on these loci.

## 1 INTRODUCTION

Many species show a wealth of phenotypic differences between the sexes (Parsch & Ellegren, 2013). However, apart from genes on sex chromosomes, males and females share the same genome, and sexually dimorphic traits are therefore thought to arise as a result of differential regulation of genes occurring in both sexes (Ellegren & Parsch, 2007; Mank, 2017; Pointer, Harrison, Wright, & Mank, 2013; Ranz, Castillo‐Davis, Meiklejohn, & Hartl, 2003), often referred to as sex‐biased gene expression. Sex‐biased genes are thought to evolve in response to conflicting sex‐specific selection pressures over optimal expression acting on this shared genetic content (Connallon & Knowles, 2005) and are increasingly used to study the footprint of sex‐specific selection within the genome (Dean et al., 2017; Gossmann, Schmid, Grossniklaus, & Schmid, 2014; Mank, 2017).

In contrast to animals, where sexual dimorphism is more frequent, only a small percentage (~5%) of flowering plants are dioecious (Renner, 2014; Robinson et al., 2014), where individuals have exclusively male or female reproductive organs. The majority (~90%) of angiosperms are hermaphroditic (Ainsworth, 2000; Barrett & Hough, 2013), where flowers are bisexual, while another small fraction are monoecious, where separate flowers within the same plant carry different reproductive organs (Renner, 2014). Despite being rare, dioecy has evolved in flowering plants many times independently (Charlesworth, 2002) and is distributed across the majority of angiosperm higher taxa (Heilbuth, 2000; Käfer, Marais, & Pannell, 2017).

Although sexual dimorphism is generally more extensive in animal species, male and female dioecious flowering plants also undergo conflicts over trait optima and are subject to natural and sexual selection leading to a range of phenotypic sexual differences (Barrett & Hough, 2013). Studies of differential male and female gene expression patterns in plants (Muyle, Shearn, & Marais, 2017) indicate that sex‐biased gene expression plays a role in the evolution of sexual dimorphism in morphological (e.g., anther and ovule development pathways in asparagus, Harkess et al., 2015), physiological (e.g., salinity tolerance in poplars, Jiang et al., 2012) and ecological traits (e.g., response to fungal infection in *Silene latifolia*, Zemp, Tavares, & Widmer, 2015).

Extensive studies in plants and animals have shown that genes with sex‐biased expression vary in abundance across different developmental stages and tissues (Grath & Parsch, 2016; Perry, Harrison, & Mank, 2014; Robinson et al., 2014; Zemp et al., 2016; Zluvova, Zak, Janousek, & Vyskot, 2010). Evolutionary dynamics analyses also indicate that different evolutionary pressures impact the rate of sequence evolution of sex‐biased genes; for example, sex‐biased genes in reproductive tissues tend to have different rates of protein evolution compared to unbiased genes (Dean et al., 2017; Lipinska et al., 2015; Mank, Nam, Brunström, & Ellegren, 2010a; Perry et al., 2014; Sharma et al., 2014). In animal systems, where the rates of sequence divergence of sex‐biased genes have been studied more widely, male‐biased genes in many species, including *Drosophila* and adult birds, tend to be more numerous and to have higher expression and divergence rates (Assis, Zhou, & Bachtrog, 2012; Grath & Parsch, 2016; Harrison et al., 2015; Khaitovich et al., 2005) compared to female‐biased and unbiased genes. This has often been interpreted as the signature of sexual selection, particularly sperm competition (Ellegren & Parsch, 2007). However, studies in other organisms have reported elevated rates of evolution in female‐biased genes (Mank et al., 2010a; Whittle & Johannesson, 2013), leading to questions about the relationship between rates of evolution and sexual selection. In *Arabidopsis*, genes expressed in pollen have lower rates of evolution (Gossmann et al., 2014). Moreover, nonadaptive evolutionary processes have been shown to drive the fast rates of sequence evolution observed in sex‐biased genes in some systems (Gershoni & Pietrokovski, 2014; Harrison et al., 2015) perhaps related to relaxed purifying selection (Hunt et al., 2011).

Sexual selection in flowering plants is also thought to be strong (Moore & Pannell, 2011), acting on gene expression patterns predominantly through pollen competition. Male gametophytic tissue in *Arabidopsis thaliana* and rice has been shown to express a higher proportion of recently evolved genes compared to other tissues (Cui et al., 2015). Some of these young genes possess essential pollen‐specific functions, suggesting a role for pollen competition in facilitating de novo gene development. As male‐biased mutation is thought to be strong due to the elevated numbers of germ cell divisions in male cells (Whittle & Johnston, 2003), pollen competition, in this case, was suggested to counteract the potentially negative effects of higher mutation rates present in male gametophytes (Cui et al., 2015). Similarly, younger genes in the gametophyte of *A. thaliana*, rice and soya bean were also found to have higher rates of evolution compared to genes in the sporophytic tissue, however to varying degrees in males and females (Gossmann, Saleh, Schmid, Spence, & Schmid, 2016). Suggested reasons for these findings concerned the lower tissue complexity, and hence lower genetic interaction, in the gametophyte as well as differences between the sexes.

Plants additionally differ from animals in having a longer haploid phase in their life cycle, suggesting that haploid selection may act more forcefully to remove mildly deleterious recessive variation in pollen‐expressed genes. Previous work on *A. thaliana* showed that the predominance of selfing, and similarly the intragametophytic selfing in moss species (Szövényi et al., 2014), leads to the more effective purging of mildly deleterious recessive variation (Gossmann et al., 2014). In the obligate outcrossing plant *Capsella grandiflora*, pollen‐specific genes, but not sperm‐enriched genes, evolve under both stronger purifying and positive selection compared to genes from sporophytic tissues (Arunkumar, Josephs, Williamson, & Wright, 2013). These findings are indicative of a potential combined effect of haploid selection and pollen competition acting in pollen‐specific cells, whereas selective pressures are expected to be more relaxed for sperm‐specific genes as there is no competition between them (Arunkumar et al., 2013).

These studies make it increasingly clear that many evolutionary forces shape the sequence evolution of sex‐biased genes, including sexual selection through sperm competition (Ellegren & Parsch, 2007), haploid selection and natural selection (Ingvarsson, 2010). Particularly in plants, in order to understand the relative contribution of these forces, it is important to study rates of evolution in species with different levels of gamete competition, motivating studies on outcrossing dioecious species.

**Figure 1:**
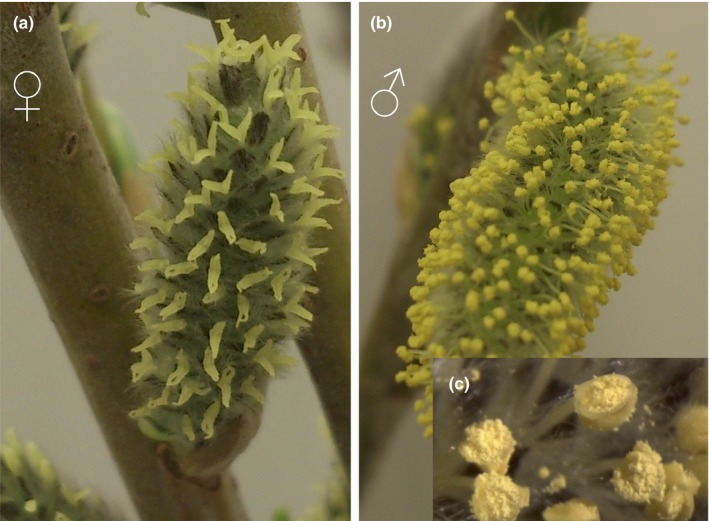
Physical appearance of adult *S. viminalis* catkins. (a) Female catkins with protruding pistillate flowers. (b) Male catkins with protruding staminate flowers. (c) Anthers of male catkins abundant in pollen grains

The basket willow, *Salix viminalis*, is a dioecious woody angiosperm (Cronk, Needham, & Rudall, 2015), belonging, together with other willow and poplar (*Populus*) species, to the Salicaceae family. *S. viminalis* is characterized by rapid seed development and growth (Ghelardini et al., 2014); it is both insect‐ and wind‐pollinated (Peeters & Totland, 1999); and it has a recently evolved ZW sex chromosome system (Pucholt, Wright, Conze, Mank, & Berlin, 2017). Willow and poplar species have reproductive structures characterized by clusters of unisexual inflorescences referred to as catkins (Figure 1). Flowers in male willow catkins present a reduced number of stamens with anthers and filaments; however, they lack a vestigial ovary, indicating floral reduction compared to other related non‐catkin‐bearing dioecious species (Cronk et al., 2015; Fisher, 1928). Flowers in female willow catkins contain pistils with style, stigma and an ovary. However, they also show a high degree of floral reduction as there is an absence of staminodes and, similarly to male catkins, they lack a perianth with petals and sepals (Cronk et al., 2015; Fisher, 1928; Karrenberg, Kollmann, & Edwards, 2002), potentially with a role in facilitating wind pollination (Karrenberg et al., 2002).

Our study of gene expression patterns in male and female *S. viminalis* individuals begins to explore the selective forces acting on sex‐biased gene evolution in dioecious plants. We analysed sex‐biased gene expression patterns in *S. viminalis* from two different tissues, vegetative (leaf) and sex‐specific reproductive (catkin) tissue. We found the reproductive tissue to be more transcriptionally dimorphic and identified overall higher expression levels for male‐biased genes than for female‐biased genes, consistent with previous studies (Grath & Parsch, 2016). Interestingly, however, we found that in catkin, male‐biased genes on the autosomes and the pseudoautosomal region have significantly lower rates of sequence divergence than both unbiased and female‐biased genes. Similarly, female‐biased genes show lower rates of sequence evolution in comparison with unbiased genes; however, the difference is not significant. We could not detect any significant differences in the proportion of genes evolving under positive selection between either male‐biased or female‐biased genes and unbiased genes. The low rates of male‐biased sequence evolution could be partly explained by the higher rate of silent mutations in male‐biased genes resulting from lower codon usage bias. However, haploid selection would also be expected in this tissue to exert a stronger purifying force to remove deleterious recessive mutations.

## 2 MATERIALS AND METHODS

### 2.1 Sample collection and sequencing

We obtained RNA‐seq data from leaves and catkins from three female (78021, 78195, 78183) and three male (81084, T76, Hallstad 1‐84) *S. viminalis* accessions (Pucholt et al., 2017; reads are deposited in the European Nucleotide Archive under Accession no. PRJEB15050). These accessions represent unrelated germplasm samples collected in Europe and Western Russia that were subsequently planted in a field archive near Uppsala, Sweden, where they were part of the *S. viminalis* association mapping population (Berlin et al., 2014; Hallingbäck et al., 2016). As previously described (Pucholt et al., 2017), stem cuttings were collected in the field and transferred to a growth chamber with 22°C constant temperature and 18 hr day length. After seven and thirteen days, respectively, fully developed adult catkins and young leaves were collected from each accession. RNA from each accession and tissue was extracted using the Spectrum Plant Total RNA Kit (Sigma‐Aldrich Co. LLC) following variant B of the instructions provided by the manufacturer and including an on‐column DNase treatment step. One RNA‐seq library for each sample was prepared from 1 μg total RNA using the TruSeq stranded mRNA sample preparation kit (Cat# RS‐122‐2101/2102, Illumina Inc.) including polyA selection. The library preparation was carried out according to the manufacturer's protocol (#15031047, rev E). Sequencing was performed on an Illumina HiSeq2500 instrument with paired‐end 125 bp read length, v4 sequencing chemistry, and all twelve libraries were pooled and sequenced on three lanes. Preparation of the RNA‐seq libraries and sequencing were performed at the SNP&SEQ Technology Platform in Uppsala, Sweden.

We recovered an average of 42 million 125‐bp paired‐end reads per sample. After assessing data quality with fastqc v0.11.3 (http://www.bioinformatics.babraham.ac.uk/projects/fastqc/), we used trimmomatic v0.36 (Lohse et al., 2012) to remove adaptor sequences and trim the reads, removing regions where the average Phred score in sliding windows of four bases was <15 as well as reads for which the leading/trailing bases had a Phred score <3. Following trimming, we removed paired‐end reads where either read pair was <50 bp (Table S1), resulting in an average of 30 million paired‐end reads per sample.

### 2.2 Expression analysis

We mapped RNA‐seq reads to the de novo male genome assembly (Pucholt et al., 2017) using hisat2 v2.0.4 (Kim, Langmead, & Salzberg, 2015), filtering reads with unpaired (‐no‐mixed option) or discordant (‐no‐discordant option) alignments. To generate a reference transcriptome, we sorted and converted alignment output sam files into bam files using samtools v1.2 (Li et al., 2009) and extracted gene coordinates for each sample using stringtie v1.2.4 (Pertea et al., 2015) with default parameters. We then merged output GTF files of all samples to obtain a nonredundant set of transcript coordinates and used bedtools getfasta to extract sequences (Quinlan & Hall, 2010). We filtered ncRNA by BLASTing transcript sequences to the *Arabidopsis thaliana* ncRNA (Ensembl Plants 32; Flicek et al., 2014) using blastn and an e‐value cut‐off of 1 × 10^−10^.

We extracted read alignments for transcripts in each sample and tissue separately from the filtered transcriptome reference using stringtie and obtained read counts using htseq v.0.6.1 (Anders, Pyl, & Huber, 2015). RPKM values were estimated using edger (Robinson, McCarthy, & Smyth, 2010) in r (R core team 2015) and transcripts filtered for a minimum expression threshold of 2 RPKM in at least half of the individuals in one sex (in this case, at least two of the three individuals per each sex) as per previous similar studies (Harrison et al., 2015; Pointer et al., 2013). We only retained transcripts with positional information on annotated chromosomes (Pucholt et al., 2017) for further analysis and normalized separately for each tissue using TMM in edger.

We performed hierarchical clustering of average gene expression for genes expressed in both tissues with bootstrap resampling (1,000 replicates) in the r package pvclust v.2.0 (R Core Team, 2015; Suzuki & Shimodaira, 2006). We generated a heatmap of log_2_ average male and female expression in the two tissues using the r package pheatmap v.1.0.7 (Kolde, 2012; R Core Team, 2015).

We identified sex‐biased expression based on a minimum of twofold differential expression (log_2_ M:F RPKM > 1 for male‐biased expression and < −1 for female‐biased expression) and a significant *p* value (*p*_adj _< .05 following FDR correction for multiple testing (Benjamini & Hochberg, 1995)) in edger.

### 2.3 Sequence divergence analysis

Additional to *S. viminalis*, we obtained coding sequences for *P. trichocarpa* from Ensembl Plants 32 (Flicek et al., 2014), *Populus tremula* and *Populus tremuloides* from PopGenIE (Sundell et al., 2015) and *Salix suchowensis* (http://115.29.234.170/willow/ (Dai et al., 2014)). The longest transcript for each gene was identified in all species, and a reciprocal blastn with an e‐value cut‐off of 1 × 10^−10^ and a minimum percentage identity of 30% was used to identify orthologs. We used blastx to obtain open reading frames of the identified orthologous groups, which we aligned with prank v140603 (Löytynoja & Goldman, 2008), using the rooted tree ((*Salixviminalis, Salixsuchowensis*), ((*Populustremula*,* Populustremuloides*), *Populustrichocarpa))*. Gaps were removed from the alignments.

To ensure the accurate calculation of divergence estimates, poorly aligned regions were masked with swamp v 31‐03‐14 (Harrison, Jordan, & Montgomery, 2014). We employed a two‐step masking approach, first using a shorter window size to exclude sequencing errors causing short stretches of nonsynonymous substitutions and then a large window size to remove alignment errors caused by variation in exon splicing (Harrison et al., 2014). Specifically, we first masked regions with more than seven nonsynonymous substitutions in a sliding window scan of 15 codons, followed by a second masking where more than two nonsynonymous substitutions were present in a sliding window scan of four codons. To choose these thresholds, we imposed a range of masking criteria on our data set and conducted the branch‐site test on these test data sets. We manually observed the alignment of genes with the highest log likelihood scores to choose the most efficient and appropriate masking criteria. We subsequently removed genes where the alignment (after removal of gaps and masked regions) was < 300 bp, which likely represent incomplete sequences. This resulted in 7,631 1:1 orthologs.

We tested the robustness of the 1:1 orthologs data set (Supporting Information) by separately inferring orthologous groups using orthomcl (Li, Stoeckert, & Roos, 2003), an approach with higher specificity (Altenhoff & Dessimoz, 2009). As orthomcl relies on the Markov Clustering algorithm, it is useful in identifying cases of co‐orthology (a duplicate of a gene in one species that is orthologous to a gene in another species) within the total 1:1 orthologous groups identified. By excluding these co‐orthologous groups, we recovered fewer 1:1 orthologs (1,346 after filtering for polymorphism and divergence data); however, the divergence results were consistent with our broader data set based on reciprocal blast (Table S2). As such, we concluded that the reciprocal best‐hit approach was appropriate to use in this case.

We further used branch model 2 (model = 2, nssites = 0, fixomega = 0, omega = 0.4) from the CODEML package in paml v4.8 (Yang, 2007) to obtain divergence estimates and calculate mean *d*_N_/*d*_S_ specifically for the *S. viminalis* branch using the unrooted tree ((*Salixviminalis*,* Salixsuchowensis*), *Populustrichocarpa*,* Populustremula*,* Populustremuloides*). Mutation‐saturated sites did not have an effect on the resulting divergence estimates as none of the orthologs had *d*_S_ > 2 (Axelsson et al., 2008). In addition, we also obtained omega values for each sex‐bias gene category by running the CODEML branch model 2 in paml separately on the concatenated sequences of all genes in each gene category. This approach reduces the influence of codon bias in estimating rates of divergence (Bierne & Eyre‐Walker, 2003).

Based on their genomic location in the *S. viminalis* genome (Pucholt et al., 2017), we divided orthologs into two groups, orthologs on the autosomes (including the pseudoautosomal region of the Z chromosome) and orthologs on the Z‐linked nonrecombining region. Because genes on sex chromosomes can exhibit accelerated rates of evolution (Charlesworth, Coyne, & Barton, 1987), and this may be more often due to nonadaptive processes on Z chromosomes (Mank, Vicoso, Berlin, & Charlesworth, 2010b; Wright et al., 2015), we analysed rates of evolution separately for autosomal and Z‐linked loci. Mean *d*_N_ (the number of nonsynonymous substitutions over nonsynonymous sites) and mean *d*_S_ (the number of synonymous substitutions over synonymous sites) were calculated separately for each group of orthologs as the ratio of the sum of the number of substitutions across all orthologs in that group, resulted from paml, to the number of sites (*d*_N_ = sum *D*_N_/sum N; *d*_S_ = sum *D*_S_/sum S). By calculating mean *d*_N_ and *d*_S_ through this method, the issue of infinitely high *d*_N_/*d*_S_ estimates arising from low *d*_S_ sequences and skew from short sequences is avoided (Mank, Hultin‐Rosenberg, Axelsson, & Ellegren, 2007). Bootstrapping with 1,000 replicates was used to determine the 95% confidence intervals. Pairwise comparisons with 1,000 permutation test replicates were used to identify significant differences in *d*_N_, *d*_S_ and *d*_N_/*d*_S_ between the categories.

### 2.4 Polymorphism analysis

We obtained polymorphism data by mapping the RNA‐seq reads to the reference genome assembly using star aligner v2.5.2b (Dobin et al., 2013) in the two‐pass mode and with default parameters, retaining uniquely mapping reads only. We conducted SNP calling using samtools mpileup and varscan v2.3.9 mpileup2snp (Koboldt et al., 2012). We ran samtools mpileup with a maximum read depth of 10,000,000 and minimum base quality of 20 for consistency with varscan minimum coverage filtering. The base alignment quality (BAQ) adjustment was disabled in samtools as it imposes a too stringent adjustment of base quality scores (Koboldt, Larson, & Wilson, 2014). We ran varscan mpileup2snp with minimum coverage of 20, minimum of three supporting reads, minimum average quality of 20, minimum variant allele frequency of 0.15, minimum frequency for homozygote of 0.85, strand filter on and *p* value of .05. We defined valid SNPs as sites with a coverage ≥ 20 in at least half of the individuals in one sex (minimum of two of the three individuals in a sex) and a minor allele frequency ≥ 0.20, identifying a total of 235,106 SNPs. We identified whether SNPs were synonymous or nonsynonymous by matching them to the reading frame.

As the divergence and polymorphism analyses use different filtering criteria, we ensured the two data sets were comparable by identifying a set of codons where all sites pass the filtering criteria for both analyses. We only kept codons where (i) all sites pass the minimum coverage threshold of 20 in at least half of the individuals in one sex, (ii) there are no alignment gaps following prank alignment, and (iii) there were no ambiguity data (*N*_s_) following swamp masking. Only genes with both divergence and polymorphism information were used in further analyses. This ensures that the number of synonymous (S) and nonsynonymous sites (N) is identical across divergence and polymorphism analyses, and therefore suitable for McDonald–Kreitman tests. We have therefore used the same number of nonsynonymous (N) and synonymous (S) sites in our calculations of *d*_N_, *p*_N_ and, respectively, *d*_S_ and *p*_S._

We calculated mean *p*_N_ (number of nonsynonymous polymorphisms over nonsynonymous sites) and mean *p*_S_ (number of synonymous polymorphisms over synonymous sites) for each gene category as the ratio of the sum of the number of polymorphisms to the sum of the number of sites (*p*_N_ = sum *P*_N_/sum N; *p*_S_ = sum *P*_S_/sum S).

### 2.5 Analysis of synonymous codon usage bias

Codon usage bias was estimated using codonw (http://codonw.sourceforge.net) through the effective number of codons (ENC) (Wright, 1990). The ENC measure determines the degree to which the entire genetic code is used in each gene, ENC values ranging from 20 (indicating extreme bias, where only one codon is used for one amino acid) to 61 (indicating no bias, where all amino acids are represented equally by all possible codons) (Wright, 1990). This measure is not biased by the different lengths of the coding regions being analysed, and as such, it has been shown to be more reliable than other commonly used methods of estimating codon usage bias (Comeron & Aguadé, 1998). The effective number of codons was calculated for all the genes with divergence and polymorphism data (Table 2).

### 2.6 Tests of positive selection

To identify genes evolving under adaptive evolution, we used the McDonald–Kreitman test (McDonald & Kreitman, 1991), which contrasts the ratio of nonsynonymous and synonymous substitutions with polymorphisms. For each gene, we used a 2 × 2 contingency table and a Fisher's exact test in r to test for deviations from neutrality using numbers of nonsynonymous and synonymous substitutions (*D*_N_, *D*_S_) and polymorphisms (*P*_N_, *P*_S_). As the McDonald–Kreitman test lacks power with low table cell counts, genes were excluded from the analysis if, within the contingency table, the sum over any row or column was less than six (Andolfatto, 2008; Begun et al., 2007). For genes with significant deviations in *D*_N_, *D*_S_, *P*_N_ and *P*_S_, a higher nonsynonymous‐to‐synonymous substitutions ratio relative to polymorphisms ratio (*d*_N_/*d*_S_ > *p*_N_/*p*_S_) represented a signature of positive selection. We then tested for significant differences between sex‐biased and unbiased genes in the proportion of genes with signatures of positive selection using Fisher's exact test.

For each gene category, we also used the divergence and polymorphism data to calculate the average direction of selection (DoS) statistic (Stoletzki & Eyre‐Walker, 2011). DoS was calculated for each gene as the difference between the proportion of nonsynonymous substitutions and the proportion of nonsynonymous polymorphisms (DoS = *D*_N_/(*D*_N_ + *D*_S_) − *P*_N_/(*P*_N_ + *P*_S_)), where positive DoS values indicate positive selection, a value of zero indicates neutral evolution while negative values indicate purifying selection and segregating deleterious mutations (Stoletzki & Eyre‐Walker, 2011). Additional to the McDonald–Kreitman test, we also used the DoS statistic to test, using Fisher's exact test, for differences in the proportion of fixed nonsynonymous sites and nonsynonymous polymorphisms.

## 3 RESULTS

### 3.1 Gene expression in catkin and leaf

RNA‐seq reads from two tissues, catkin (reproductive tissue) and leaf (vegetative tissue), of male and female *S. viminalis* individuals were mapped to the genome assembly yielding an average of 30 million read mappings per sample after quality control and trimming (Table S1). Following expression filtering, we recovered 8,186 significantly expressed genes in catkin and 7,638 significantly expressed genes in leaf.

**Figure 2:**
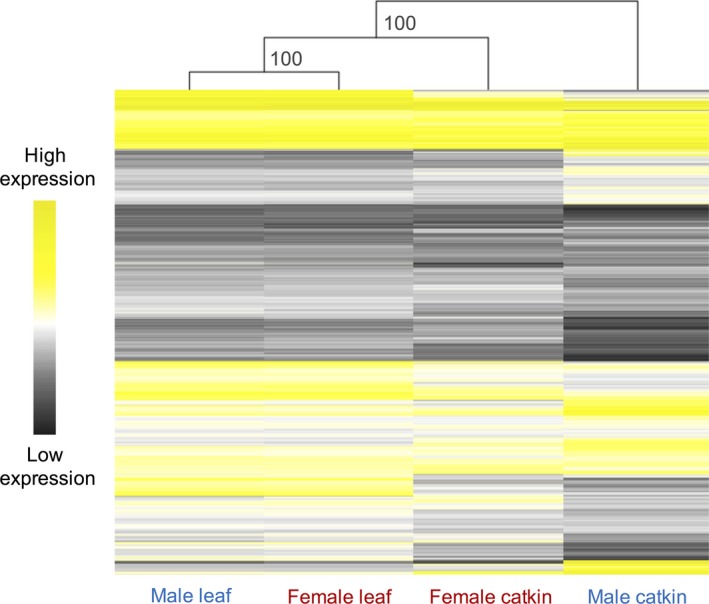
Heatmap and hierarchical clustering of average male (blue) and average female (red) gene expression in catkin and leaf. The heatmap represents all the filtered genes expressed in both tissues (7,257). Hierarchical gene clustering is based on Euclidean distance with average linkage for log_2_ RPKM expression for each gene. Numbers at nodes represent the 1,000 replicates percentage bootstrap results

**Figure 3:**
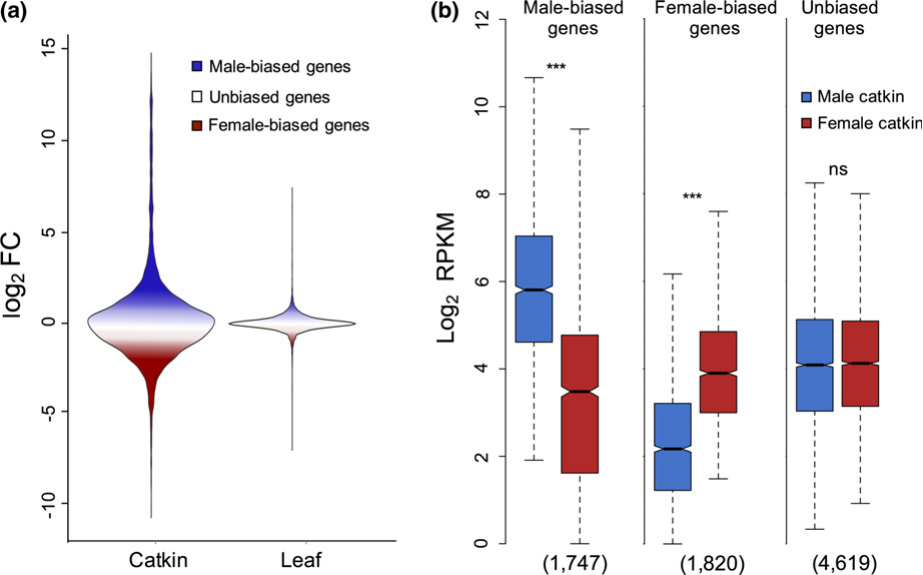
Sex‐biased gene expression in *Salix viminalis*. (a) Proportion and range of differentially expressed and unbiased genes in catkin and leaf. (b) Comparison between male and female average expression for sex‐biased and unbiased genes in catkin. Numbers in brackets represent the number of genes in each category. Significant differences between male and female expression based on Wilcoxon rank sum tests are denoted (ns = nonsignificant, ***p <* *.001)

We first assessed transcriptional similarity across tissues and sexes using hierarchical clustering of gene expression levels (Figure 2). We found that the reproductive tissue was more transcriptionally dimorphic than the vegetative tissue, consistent with studies in many other species (Jiang & Machado, 2009; Mank, Hultin‐Rosenberg, Webster, & Ellegren, 2008; Pointer et al., 2013; Yang, Zhang, & He, 2016). Expression for male catkin clustered most distantly from both male and female expressions in leaf. We identified 3,567 genes (43% of all filtered catkin genes) showing sex‐biased expression in catkin (log_2_ fold change > 1 or < −1, *p*_adj_ < .05), compared to expression in the vegetative tissue, where we identified only seven (0.09%) leaf sex‐biased genes (Figure 3). Even with a more relaxed fold change threshold for defining differentially expressed genes (log_2_ fold change > 0.5 or < −0.5, *p*_adj_ < .05), we still could not identify any additional leaf sex‐biased genes. There were also no shared sex‐biased genes between the two tissues.

### 3.2 Dynamics of catkin sex‐biased gene expression

Although female‐biased genes (*n* = 1,820) were slightly more numerous than male‐biased genes (*n* = 1,747), the magnitude of differential expression (log_2_ FC) for male‐biased genes was significantly greater than that for female‐biased genes (Wilcoxon rank sum test *p *<* *.001). Average male expression for male‐biased genes was significantly higher than average female expression for female‐biased genes (Figure 3, Wilcoxon rank sum test *p *<* *.001), although male expression for female‐biased genes was significantly lower than female expression for male‐biased genes (Figure 3, Wilcoxon rank sum test *p *<* *.001).

**Figure 4:**
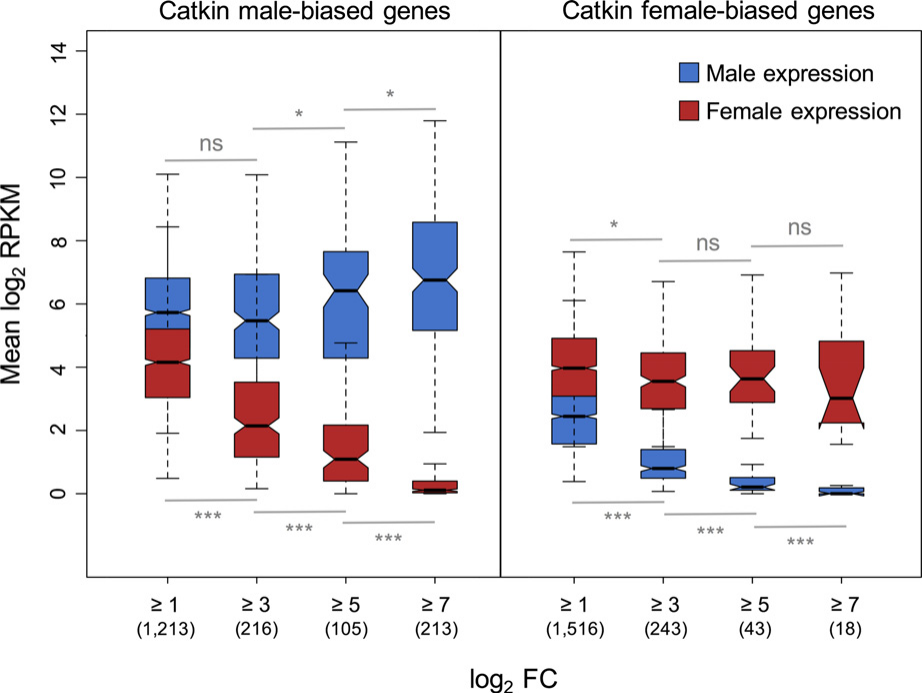
Average male and female catkin gene expression at different sex‐bias fold change thresholds for all assessed catkin male‐biased and female‐biased genes. Numbers in brackets represent the number of genes in each fold change category. Significance level is based on Wilcoxon rank sum tests (ns = nonsignificant, *p <* *.05, ***p <* *.001)

We grouped sex‐biased genes based on different fold change thresholds and compared average male and female catkin expression for the genes in each category. This analysis suggests that catkin male‐biased genes may arise from increased expression in males and decreased expression in females (Figure 4). For female‐biased genes, however, there is a decreasing trend in male expression with increasing fold change thresholds but a constant female expression across all thresholds (Figure 4), suggesting that female bias results primarily from downregulation of male expression.

The paucity of sex‐biased genes in the leaf tissue makes it a useful comparison to further assess the sex‐specific changes that give rise to male‐ and female‐biased genes. We therefore used leaf expression as the putative ancestral expression state. For the subset of catkin sex‐biased genes that also had expression in the leaf tissue, we determined the difference in expression between catkin and leaf across the same fold change thresholds used in Figure 4. For male‐biased genes in the catkin, we found significant differences between catkin and leaf expression in both sexes, although to a lesser extent in females (Figure S1). On the other hand, for catkin female‐biased genes, we also observed large differences in male expression between catkin and leaf samples; however, we found little to no female expression changes between the two tissues (Figure S1).

We further divided catkin sex‐biased genes into autosomal (including the pseudoautosomal region of the sex chromosomes) and Z‐linked genes. On the autosomes, we found 3,536 sex‐biased genes (1,728 male‐biased and 1,808 female‐biased genes). On the nonrecombining region of the Z chromosome, we found only 31 sex‐biased genes (19 male‐biased and 12 female‐biased genes); however, considering the narrow region of recombination suppression between the sex chromosomes (Pucholt et al., 2017, 3.5–8.8 Mbp), these sex‐biased genes represented 44% of the total identified gene content in the nonrecombining sex‐chromosome region.

### 3.3 Rates of evolution

We compared the overall ratios of nonsynonymous‐to‐synonymous nucleotide substitutions (*d*_N_/*d*_S_) between catkin and leaf and found no significant differences between the two (*p* = .476, significance based on permutation tests with 1,000 replicates). We also did not find a significant difference in the evolution of unbiased genes between the two tissues (*p* = .056 from permutation tests with 1,000 replicates), likely influenced by the large overlap of genes between them (97% of catkin unbiased genes represent 58% of the unbiased genes expressed in leaf). We found too few significantly sex‐biased genes in the leaf tissue to make any statistical comparisons of rates of sequence evolution between catkin and leaf sex‐biased genes.

We also compared the ratio of *d*_N_/*d*_S_ between sex‐biased and unbiased genes in catkin to test for differences in the rate of evolutionary divergence. Interestingly, we found that on autosomes, although male‐biased genes have more amino acid substitutions than both unbiased and female‐biased genes, as shown by significantly higher *d*_*N*_ values, *d*_N_/*d*_S_ for male‐biased genes was significantly lower, indicating slower rates of functional evolution relative to unbiased (Table 1; Table S2) and female‐biased genes (*p *<* *.001, significance based on permutation tests with 1,000 replicates). Similar results were obtained when we estimated *d*_N_/*d*_S_ from a data set of 1:1 orthologs that excluded cases of co‐orthology (Table S2), as well as from omega values resulting from running CODEML branch model 2 in paml on concatenated sequences of genes in each sex‐bias gene category (Table S3). This lower *d*_N_/*d*_S_ ratio is caused in part by a disproportionate increase in synonymous substitutions compared to nonsynonymous substitutions, causing the relationship between *d*_*N*_ and *d*_*S*_ in male‐biased genes to lie further away from direct proportionality than in the case of unbiased genes (Figure S2).

**Table 1:** Divergence and polymorphism estimates for catkin gene categories on autosomes and the nonrecombining Z region

Tissue	Location	Category^a^	n Genes^b^	*d*_N_ (95% CI) sig.^c^	*d*_S_ (95% CI) sig.^c^	*d*_N_/*d*_S_ (95% CI) sig.^c^	*p*_N_ (95% CI) sig.^c^	*p*_S_ (95% CI) sig.^c^	*p*_N_/*p*_S_ (95% CI) sig.^c^	DoS sig.^d^
Catkin	Autosomes and recombining Z	UB	1,754	0.0030 (0.0028–0.0031)	0.0135 (0.0130–0.0141)	0.2204 (0.2101–0.2311)	0.0027 (0.0025–0.0028)	0.0109 (0.0103–0.0114)	0.2456 (0.2328–0.2584)	−0.0495
		MB	674	**0.0032 (0.0030**–**0.0035)** ***p*** = **.012**	**0.0162 (0.0147**–**0.0187)** ***p *** **<** *** *** **.001**	**0.1951 (0.1769**–**0.2157)** ***p *** **<** *** *** **.001**	**0.0029 (0.0026**–**0.0032)** ***p*** = **.022**	**0.0116 (0.0108**–**0.0124)** ***p*** = **.042**	0.2491 (0.2260–0.2727) *p* = .682	−0.0346 *p* = .627
		FB	732	0.0031 (0.0029–0.0033) *p* = .094	**0.0149 (0.0141**–**0.0158)** ***p *** **<** *** *** **.001**	0.2095 (0.1938–0.2256) *p* = .082	**0.0030 (0.0028**–**0.0033)** ***p*** = **.002**	**0.0121 (0.0113**–**0.0131)** ***p *** **<** *** *** **.001**	0.2477 (0.2293–0.2666) *p* = .774	−0.0375 *p* = .916
	Nonrecombining Z	UB	12	0.0032 (0.0024–0.0043)	0.0102 (0.0056–0.0145)	0.3130 (0.2141–0.5498)	0.0015 (0.0007–0.0032)	0.0045 (0.0022–0.0078)	0.3407 (0.1778–0.5588)	0.0800
		MB	3	0.0029 (0.0–0.0140) *p* = .378	0.0143 (0.0091–0.0396) *p* = .730	0.2019 (0.0–0.3533) *p* = .244	0.0029 (0.0–0.0210) *p* = .396	0.0104 (0.0039–0.0505) *p* = .084	0.2781 (0.0–0.4151) *p* = .082	0.0088 *p* = .563
		FB	4	0.0032 (0.0021–0.0037) *p* = .964	0.0100 (0.0061–0.0207) *p* = .926	0.3172 (0.1649–0.4996) *p* = .948	**0.0045 (0.0008**–**0.0082)** ***p*** = **.026**	**0.0138 (0.0055**–**0.0229)** ***p *** **<** *** *** **.001**	0.3243 (0.0845–0.4657) *p* = .882	0.0770 *p* = .761

a Unbiased (UB), male‐biased (MB) and female‐biased (FB) genes.

b Number of genes with both divergence and polymorphism data.

c *p* values based on 1,000 replicates permutation tests comparing male‐biased and female‐biased genes with unbiased genes. Significant *p* values (< .05) are shown in bold.

d *p* values from Wilcoxon nonparametric tests comparing male‐biased and female‐biased genes with unbiased genes. Significant *p* values (< .05) are shown in bold.

Female‐biased autosomal loci also showed the same pattern as male‐biased genes relative to unbiased genes; however, this result was not significant (Table 1; Table S2). On the nonrecombining Z, male‐biased genes also show lower rates of evolution compared to unbiased genes; however, this finding was not significant, likely due to the small sample size of male‐biased genes (*n* = 3). In contrast, female‐biased Z‐linked loci showed accelerated rates of evolution in comparison with male‐biased Z‐linked genes (*p *<* *.001, significance based on permutation tests with 1,000 replicates).

Highly expressed genes are often observed to exhibit lower *d*_N_/*d*_S_ values (Cherry, 2010; Drummond, Bloom, Adami, Wilke, & Arnold, 2005; Pál, Papp, & Hurst, 2001; Slotte et al., 2011); therefore, to determine whether expression level might explain our results, we divided sex‐biased and unbiased genes into quartiles based on overall expression. As expected, we found that as gene expression level increases, the rate of sequence divergence decreases and this holds true for both sex‐biased and unbiased genes (Figure S3). To further investigate the effect of expression level on the variation in rates of sequence divergence between sex‐bias categories, we used a multiple regression analysis to predict *d*_N_/*d*_S_ results based on expression level and degree of sex‐bias. For defining the degree of sex‐bias, genes were classed into five groups, highly female‐biased genes (FC ≤ −3), lowly female‐biased genes (−3 < FC ≤ −1), unbiased genes (−1 < FC < 1), lowly male‐biased genes (1 ≤ FC < 3) and highly male‐biased genes (FC ≥ 3). We found a significant negative relationship between *d*_N_/*d*_S_ values and both average log_2_ RPKM expression level (β = −.03, *p *<* *.001) and degree of sex‐bias (β = −.04, *p* = .014). There was no significant effect of the interaction between expression level and degree of sex‐bias on *d*_N_/*d*_S_ results, suggesting that any differences in the rates of sequence evolution due to sex‐bias are independent of the gene expression level for each sex‐bias category. Despite these results, the adjusted *r* ^2^ was very low (*r* ^2^ = .01), indicating that other factors, such as purifying or haploid selection, largely explain the vast majority of sequence divergence results.

We also estimated average levels of synonymous codon usage bias for sex‐biased and unbiased genes to determine whether this could explain the differences in the rates of synonymous substitutions between the gene categories. Stronger codon usage bias has been associated with higher gene expression as selective forces act to increase translational efficiency (Duret, 2002; Ingvarsson, 2010). Codon bias has also been shown to differ between differentially expressed genes, with male‐biased genes undergoing weaker codon usage bias than female‐biased (Mank et al., 2008; Magnusson et al., 2011; however, this varied across different developmental stages; Whittle, Malik, & Krochko, 2007) and unbiased genes (Hambuch & Parsch, 2005). Additionally, greater codon bias has been estimated for genes with lower rates of synonymous substitutions (Urrutia & Hurst, 2001).

We estimated codon usage bias for genes in each category through the effective number of codons (ENC), where stronger codon bias was indicated by lower ENC values. The differences in codon bias between the different gene categories were subtle, and the gene frequency spectra for all categories were distributed towards the higher end of the effective number of codons (ENC), hence lower codon usage bias (Figure S4). However, male‐biased genes had significantly lower codon usage bias than both unbiased (Table 2) and female‐biased genes (*p *<* *.001, significance based on permutation tests with 1,000 replicates). These findings, together with the higher rates of synonymous substitutions in male‐biased genes compared to unbiased and female‐biased genes, indicate weaker purifying selection on silent mutations in male‐biased genes (Sharp & Li, 1987).

**Table 2:** Codon usage bias for catkin sex‐bias gene categories

Tissue	Location	Category	n Genes^a^	ENC^b^sig.^c^
Catkin	Autosomes and recombining Z	Unbiased	1,754	52.15
		Male biased	674	52.71 ***p *** **<** *** *** **.001**
		Female biased	732	52.20 *p* = .588

a Number of genes with both divergence and polymorphism data.

b Average effective number of codons for each gene category.

c *p* values based on 1,000 replicates permutation test comparing male‐biased and female‐biased genes relative to unbiased genes. Significant *p* values (< .05) are shown in bold.

We used polymorphism data to calculate the ratio of nonsynonymous‐to‐synonymous polymorphisms (*p*_N_/*p*_S_). Sex‐biased genes on both autosomes and the nonrecombining Z region have significantly higher nonsynonymous and synonymous polymorphism levels compared to unbiased genes; however, the *p*_N_/*p*_S_ ratio was not significantly different in either of the comparisons (Table 1). To distinguish between the selective pressures acting on sequence evolution, we used the McDonald–Kreitman test of selection, comparing the ratios of *d*_N_/*d*_S_ to *p*_N_/*p*_S_ for each gene category. Following filtering, we recovered six unbiased, one male‐biased and two female‐biased genes showing signatures of positive selection (Table 3). However, there was no significant difference in the proportion of genes evolving under positive selection between either of the gene categories (Table 3, significance denoted in table). Because the McDonald–Kreitman test is extremely conservative, we also assessed selection pressures on sex‐biased genes using the direction of selection test (Stoletzki & Eyre‐Walker, 2011). Through the DoS statistic, we recovered 681 unbiased, 262 male‐biased and 282 female‐biased genes under putative positive selection (DoS > 0), yet, consistent with the McDonald–Kreitman test, we found no significant differences in the proportion of genes evolving under positive selection (Fisher's exact test *p *>* *.9 for both female‐biased and male‐biased genes in comparison with unbiased genes). Taken together, the divergence and polymorphism analyses, through tests of positive selection, suggest that the lower rates of sequence evolution seen in male‐biased genes could be due to purifying selection acting to remove deleterious recessive mutations.

**Table 3:** McDonald–Kreitman test of selection

Tissue	Location	Category	*n* Genes^a^	Positive selection^b^sig.^c^
Catkin	Autosomes and recombining Z	Unbiased	1,766	6
		Male biased	677	1 ns
		Female biased	736	2 ns

a Number of genes with both divergence and polymorphism data.

b Number of genes with significant positive selection indicated by significant deviations in *D*_N_, *D*_S_, *P*_N_ and *P*_S_ and *d*_N_/*d*_S_ > *p*_N_/*p*_S_.

c Significance based on Fisher's exact test comparing sex‐biased to unbiased genes (ns = nonsignificant).

## 4 DISCUSSION

The evolution of sex‐biased gene sequence has been extensively analysed in animal systems. In contrast, far less is known about the evolution of sex‐biased genes in plants in general and in dioecious angiosperms in particular. Previous work in *A. thaliana*, an annual and largely selfing hermaphroditic species, found low rates of evolution in pollen‐expressed genes, although with evidence of a higher proportion of sites under positive selection (Gossmann et al., 2014). This could be the result of the greater haploid selection in plants; however, it could also be, at least partially, the result of the selfing mating system in this species, which leads to the purging of recessive deleterious variation. Similarly, in the self‐incompatible close relative of *A. thaliana*,* C. grandiflora*, a larger fraction of pollen‐specific genes was found to evolve under strong purifying selection and to also exhibit faster protein evolution rates compared to sporophytic genes (Arunkumar et al., 2013). This is suggested to be the result of both higher pollen competition and the haploid nature of the pollen‐specific tissue.

Here, we investigate the evolution of sex‐biased genes in *S. viminalis*, a perennial dioecious (obligate outcrossing) species with partial wind pollination. Similarly to *C. grandiflora* (Kao & McCubbin, 1996), *S. viminalis* theoretically experiences far higher levels of pollen competition than *A. thaliana*, particularly intermale competition. Although we might expect the high levels of sperm competition in *S. viminalis* to produce higher rates of protein evolution for male‐biased genes, we observed the opposite. Moreover, in contrast to work in *C. grandiflora* (Arunkumar et al., 2013), we did not find evidence of a high proportion of male‐biased genes under positive selection.

The observed dynamics of sex‐biased gene expression in *S. viminalis* is consistent with previous reports in a wide range of species. Equivalent to studies on somatic and reproductive tissues in animal systems (Mank, 2017; Pointer et al., 2013; Yang et al., 2016), we found that the reproductive tissue was far more transcriptionally dimorphic than the vegetative tissue (Figures 2 and 3). Additionally, in plant species in particular, very few studies have been able to identify any significant sex‐biased genes in nonreproductive tissues (Robinson et al., 2014; Zemp, Minder, & Widmer, 2014; Zluvova et al., 2010). We also found that, in catkin, male‐biased genes were expressed at significantly higher levels and had a higher magnitude of sex‐bias than female‐biased genes (Figure 3). The level of sex‐biased gene expression found in the *S. viminalis* reproductive tissue is markedly lower than that in animal species (Jiang & Machado, 2009; Pointer et al., 2013), consistent with the significantly higher degree of sexual dimorphism in animal systems. On the other hand, we found a larger percentage of sex‐biased genes compared to several plant and algae species with low levels of sexual dimorphism (Harkess et al., 2015; Lipinska et al., 2015; Zemp et al., 2016). This is indicative of higher intersexual morphological differences in the *S. viminalis* reproductive tissue, which is consistent with previous descriptions of the structural differences between male and female catkins (Cronk et al., 2015).

Contrary to findings from the dioecious *Silene latifolia* (Zemp et al., 2016), however similarly to reports from animal and algae systems (Lipinska et al., 2015; Perry et al., 2014), our results indicate that sex‐biased gene expression has likely evolved as an outcome of expression changes in males (Figure S1). This would also explain why catkin male samples are more transcriptionally different than catkin female samples with respect to leaf samples (Figure 2). These results suggest that ancestral intralocus sexual conflict may have been more detrimental to males, leading to the evolution of sex‐biased gene expression in order to resolve such conflicts.

Additionally, although not statistically significant, we found that male‐biased genes had higher *p*_N_/*p*_S_ values compared to both unbiased and female‐biased genes, which is in stark contrast to divergence results where we found male‐biased genes to have significantly lower *d*_N_/*d*_S_ values. Given that perturbations in population size can alter estimates of polymorphism (Pool & Nielsen, 2007; Tajima, 1989), it is difficult to assess the causes of the contrasting results between *d*_N_/*d*_S_ and *p*_N_/*p*_S_ estimates for sex‐biased genes. Nevertheless, divergence estimates are less sensitive to demographic fluctuations and we more strongly rely on this measurement in our analyses of evolutionary rates of sex‐biased genes.

Sex‐biased genes in willow exhibit higher expression levels than unbiased genes, and highly expressed male‐biased and female‐biased genes had significantly lower rates of evolution than unbiased and lowly expressed sex‐biased genes (Figure S3). The fact that highly expressed genes evolve more slowly could be due to a range of different reasons, which are still highly debated (Drummond et al., 2005). The structural or functional features of the proteins they encode (Drummond et al., 2005), high pleiotropic constraints acting on the genes (Pál et al., 2001) as well as gene conversion events (Petes & Hill, 1988) have all been suggested as potential mechanisms through which highly expressed genes could have lower rates of sequence evolution. Although the high expression of many sex‐biased genes in *S. viminalis* may partially explain their slower rates of evolution, our analysis revealed a very weak correlation between expression level and rate of evolution, indicating that, in this case, expression level does not largely explain the low rates of sex‐biased gene evolution.

It is interesting that the lower *d*_N_/*d*_S_ values of male‐biased genes are associated with an overall increase in synonymous mutations relative to nonsynonymous mutations (Figure S2). This, plus our observation that male‐biased genes experience lower levels of codon usage bias (Table 2), could suggest that our *d*_N_/*d*_S_ results have been influenced by different levels of codon usage across gene expression categories. Different selection forces are thought to lead to codon usage bias, such as positive selection for preferred synonymous mutations (mutations that lead to preferred codons) and purifying selection acting on unfavourable mutations, preventing a decrease in the frequency of preferred codons (Hershberg & Petrov, 2008). Despite previous expectations that selection acting at synonymous sites is weak (Akashi, 1995; Hershberg & Petrov, 2008), several studies suggest that a range of selection strengths, spanning from weak to strong selection, influence the evolution of synonymous mutations, and hence codon usage bias measures (Hershberg & Petrov, 2008; Lawrie, Messer, Hershberg, & Petrov, 2013). However, although differential codon bias across expression categories has the potential to influence our *d*_N_/*d*_S_ estimates, our additional paml analysis (Table S3) indicates that this is not likely to be the case.

Similar to the findings from *A. thaliana* and *C. grandiflora*, the unusual rates of evolution of sex‐biased genes in *S. viminalis* could also be explained by the differential selection pressures acting on diploid versus haploid life stages. Haploid selection (Joseph & Kirkpatrick, 2004) is more effective at removing recessive deleterious mutations than selection in the diploid life stages, where dominant alleles can mask the effects of deleterious recessive alleles (Kondrashov & Crow, 1991). Although all predominantly diploid organisms pass through both haploid and diploid phases, animal species employ different mechanisms through which selection on the haploid stage is minimized (Otto, Scott, & Immler, 2015). Not only can aneuploid spermatids still be potentially viable (Lindsley & Grell, 1969), indicating limited haploid expression, but studies in mice have shown that genetically haploid spermatids evade haploid selection by sharing gene products through cytoplasmic bridges (Erickson, 1973), becoming thus phenotypically diploid (Braun, Behringer, Peschon, Brinster, & Palmiter, 1989).

Haploid selection is far more extensive in plants due to both the larger proportion of the life cycle spent in the haploid phase and active gene transcription, which has been observed in gametes, particularly in pollen (Otto et al., 2015). In addition to haploid selection, male gametophytes in angiosperm species are under strong sexual selection pressures (Erbar, 2003; Snow & Spira, 1996), particularly in outcrossing species. Mechanisms of sexual selection in angiosperms include pollen tube and pistil interactions and pollen competition over ovules, which is exacerbated in outcrossing species (Bernasconi et al., 2004).

It is important to note that the reduced floral structure and microscopic nature of the catkin (Cronk et al., 2015) makes it nearly impossible to separate haploid from diploid reproductive tissue in this species. However, our catkin preparations are highly enriched for haploid cells (Figure 1) when compared to the vegetative samples. We expect that rates of evolution for purely haploid sex‐biased tissue would be even lower than what we observe if haploid selection is indeed the primary cause of the slower rates of evolution.

Apart from insect pollen dispersal, willows also have wind‐dispersed pollination (Peeters & Totland, 1999) and experience high levels of pollen competition. The observed patterns of gene sequence evolution in *S. viminalis* support the notion that pollen competition in conjunction with haploid selection produces greater levels of purifying selection on male‐biased genes. This would remove deleterious variation and lead to significantly slower rates of functional gene sequence evolution. Interestingly, the algae *Ectocarpus*, a species where sex‐biased genes are subject almost entirely to haploid selection, shows accelerated rates of evolution for both male‐ and female‐biased genes (Lipinska et al., 2015). This suggests that haploid selection may not be the only force that influences the rate of evolution of sex‐biased genes in haploid cells. Indeed, data from haploid‐specific genes (pollen‐specific genes in *S. viminalis*) would help to more precisely determine the degree to which the currently observed lower rates of evolution of male‐biased genes can be explained by haploid selection or other factors such as expression breath (Arunkumar et al., 2013; Gossmann et al., 2014; Szövényi et al., 2013).

In summary, our findings are generally consistent with previous reports on the patterns of sex‐bias gene expression in plant and animal species. However, different forces may differentiate patterns of evolution between animal and plant systems. The reduction in haploid selection in animals may limit the power of purifying selection to remove mildly deleterious variation, particularly when it is largely recessive. In *S. viminalis,* we observe reduced rates of evolution for male‐biased genes, consistent with increased purifying selection from the extended haploid phase. Even though male‐biased genes show relaxed levels of codon bias, this does not seem to be a major driver of the reduced rate of evolution. Future work should focus on investigating the differences in the relative strength of haploid versus diploid selection in dioecious angiosperm species in shaping the evolution of sex‐biased genes.

## AUTHOR CONTRIBUTIONS

S.B. and J.E.M. designed the research; I.D., A.E.W. and P.P. performed the research; I.D. and A.E.W. analysed the data; I.D. and J.E.M. wrote the manuscript; and all authors revised the manuscript.

## DATA ACCESSIBILITY

Reads are deposited in the European Nucleotide Archive (http://www.ebi.ac.uk/ena) under Accession no. PRJEB15050.
