# Supplementary material for: Slow evolution of sex‐biased genes in the reproductive tissue of the dioecious plant Salix viminalis
Source: Mol Ecol. 2018 Jan 22;27(3):694–708. doi: 10.1111/mec.14466 (PMC5901004; doi:10.1111/mec.14466)
Supplement: Supplementary file 1 [file MEC-27-694-s001.pdf]

SUPPORTING INFORMATION

Manuscript title: Slow evolution of sex-biased genes in the reproductive tissue of the dioecious plant *Salix viminalis*

Authors: Iulia Darolti[1], Alison E. Wright[1,2], Pascal Pucholt[3,4], Sofia Berlin[3*], Judith E. Mank[1,5*]

Affiliations: 1 Department of Genetics, Evolution and Environment, University College London, United Kingdom; 2 Current address: Department of Animal and Plant Sciences, University of Sheffield, United Kingdom; 3 Department of Plant Biology, Swedish University of Agricultural Sciences, Sweden, Linnean Centre for Plant Biology; 4 Current address: Array and Analysis Facility, Department of Medical Science, Uppsala University, Sweden; 5 Department of Organismal Biology, Uppsala University, Sweden; * joint senior authors

MATERIALS AND METHODS

Determining 1:1 orthologs using OrthoMCL

We obtained protein sequences for *P. trichocarpa* from Ensembl Plants 32 (Flicek et al., 2014) and for *P. tremula* and *P. tremuloides* from PopGenIE (Sundell et al., 2015). As the protein sequences for *S. suchowensis* were not available, we used AUGUSTUS (Stanke and Morgenstern, 2005) to obtain the protein sequences for *S. suchowensis* and *S. viminalis* from the coding sequences of the longest transcripts of their genes. We ran AUGUSTUS with default parameters, *Arabidopsis thaliana* as the reference species and with the option of only predicting complete genes.

We used the protein sequences for the five species to estimate 1:1 orthologs using OrthoMCL (Li et al., 2003), following the user specifications. A total of 23,776 orthologous groups were identified which we filtered to retain only groups with 1:1 orthology and with both divergence and polymorphism data, resulting in 1,346 orthologs.

RESULTS

We divided the filtered orthologs into male-biased, female-biased and unbiased genes on the autosomes (including the sex chromosome pseudoautosomal region) and the non-recombining sex chromosome region. Compared to the number of orthologs resulting from the reciprocal BLASTn, we recovered less than half of the orthologs in each category (Table S2, Table 1). Despite the lower statistical power, we observe similar divergence and polymorphism trends between the two datasets (Table S2, Table 1). Therefore, we conclude that the extended orthologous gene dataset is reliable and we perform all our subsequent analyses on that dataset.

We also identified *S. viminalis* gene duplicate pairs in order to determine whether our estimates of rates of evolution were influenced by gene duplication. We analysed *S. viminalis* duplicate genes identified by OrthoMCL that had strictly one ortholog from each of the other four species, and whose orthologs did not have any identified duplicates themselves. In total, we detected 36 duplicate gene pairs out of which only 14 pairs contained at least one of the genes with sex-biased gene expression and 3 pairs with both members exhibiting sex-biased expression. None of the duplicate gene pairs had both members within the pair present in our divergence and polymorphism analyses as at least one of the members had been previously removed from the dataset through the different filtering criteria used. Therefore, we can conclude that the estimates of sequence divergence and polymorphism are not influenced by gene duplication.

REFERENCES

Flicek, P., Amode, M. R., Barrell, D., Beal, K., Billis, K., Brent, S., ... Searle, S. M. J. (2014). Ensembl 2014. *Nucleic Acids Res., 42*, 749-755. doi:10.1093/nar/gkt1196

Li, L., Stoeckert, C. J., & Roos, D. S. (2003). OrthoMCL: Identification of ortholog groups for eukaryotic genomes. *Genome Res., 13*, 2178-2189. doi: 10.1101/gr.1224503

Stanke, M., Morgenstern, B. (2005). AUGUSTUS: A web server for gene prediction in eukaryotes that allows user-defined constraints. *Nucleic Acids Res., 33,* W465-W467. doi:10.1093/nar/gki458

Sundell, D., Mannapperuma, C., Netotea, S., Delhomme, N., Lin, Y. C., Sjödin, A., ... Street, N. R. (2015). The plant genome integrative explorer resource: PlantGenIE.org. *New Phytol., 208*, 1149-1156. doi:10.1111/nph.1355

TABLES

Table S1. Sequencing and quality trimming information for each sample

Sample	Raw paired reads	Paired reads following trimming	% reads kept
78021_female_catkin	48,479,402	37,549,325	77.45
78183_female_catkin	32,434,747	23,739,771	73.19
78195_female_catkin	44,612,862	31,372,609	70.32
81084_male_catkin	36,261,721	26,799,852	73.91
Hallstad1-84_male_catkin	47,186,936	34,726,235	73.59
T76_male_catkin	49,433,319	35,279,702	71.37
78021_female_leaf	28,577,122	20,738,684	72.57
78183_female_leaf	42,774,423	31,112,678	72.74
78195_female_leaf	46,605,756	33,091,822	71.00
81084_male_leaf	42,739,744	29,762,943	69.64
Hallstad1-84_male_leaf	42,597,909	30,932,365	72.61
T76_male_leaf	45,688,342	31,098,461	68.07

Table S2. Divergence and polymorphism estimates for orthologs resulting from the OrthoMCL pipeline

Tissue	Location	Category[a]	n Genes[b]	d_N (95% CI) sig.[c]	d_S (95% CI) sig.[c]	d_N/d_S (95% CI) sig.[c]	p_N (95% CI) sig.[c]	p_S (95% CI) sig.[c]	p_N/p_S (95% CI) sig.[c]	DoS sig.[d]
Catkin	Autosomes and recombining Z	UB	738	0.0030 (0.0028 - 0.0033)	0.0130 (0.0122 - 0.0140)	0.2323 (0.2143 - 0.2518)	0.0028 (0.0026 - 0.0031)	0.0110 (0.0102 - 0.0119)	0.2571 (0.2337 - 0.2808)	-0.0495
		MB	257	0.0031 (0.0028 - 0.0035) *P* = 0.598	**0.0150 (0.0132 - 0.0170) *P* = 0.002**	**0.2065 (0.1782 - 0.2423) *P* = 0.038**	0.0030 (0.0026 - 0.0034) *P* = 0.306	0.0114 (0.0102 - 0.0128) *P* = 0.452	0.2633 (0.2269 - 0.3058) *P* = 0.688	-0.0346 *P* = 0.826
		FB	341	0.0031 (0.0028 - 0.0035) *P* = 0.458	**0.0143 (0.0131 - 0.0157) *P* = 0.004**	0.2170 (0.1941 - 0.2444) *P* = 0.132	**0.0032 (0.0028 - 0.0036) *P* = 0.014**	0.0117 (0.0105 - 0.0130) *P* = 0.096	0.2708 (0.2458 - 0.3012) *P* = 0.282	-0.0375 *P* = 0.086
	Non-recombining Z	UB	5	0.0030 (0.0016 - 0.0043)	0.0072 (0.0025 - 0.0106)	0.4093 (0.2318 - 0.8811)	0.0006 (0.0002 - 0.0010)	0.0031 (0.0012 - 0.0052)	0.1995 (0.0579 - 0.6606)	0.0800
		MB	3	0.0029 (0.0 - 0.0140) *P* = 0.756	**0.0143 (0.0091 - 0.0396) *P* < 0.001**	**0.2019 (0.0 - 0.3533) *P* < 0.001**	**0.0029 (0.0 - 0.0210) *P* < 0.001**	**0.0104 (0.0039 - 0.0505) *P* < 0.001**	0.2781 (0.0 - 0.4151) *P* = 0.592	0.0088 *P* = 0.786
		FB	2	0.0036 (0.0033 - 0.0037) *P* = 0.228	**0.0147 (0.0094 - 0.0345) *P* < 0.001**	0.2458 (0.0966 - 0.3981) *P* = 0.182	**0.0082 (0.0033 - 0.0100) *P* < 0.001**	**0.0229 (0.0226 - 0.0238) *P* < 0.001**	0.3563 (0.14 - 0.4398) *P* = 0.434	0.0770 *P* = 0.857

[a] Unbiased (UB), male-biased (MB) and female-biased (FB) genes.

[b] Number of genes with both divergence and polymorphism data.

[c] *P* values based on 1,000 replicates permutation tests comparing male-biased and female-biased genes with unbiased genes. Significant *P* values (< 0.05) are shown in bold.

[d] *P* values from Wilcoxon nonparametric tests comparing male-biased and female-biased genes with unbiased genes. Significant *P* values (< 0.05) are shown in bold.

Table S3. Comparison of divergence results from two methods of estimating d_N/d_S

				Ratio of the sum of the number of substitutions to the number of sites			Concatenated sequences		
Tissue	Location	Category[a]	n Genes[b]	d_N (95% CI) sig.[c]	d_S (95% CI) sig.[c]	d_N/d_S (95% CI) sig.[c]	d_N (95% CI) sig.[c]	d_S (95% CI) sig.[c]	d_N/d_S (95% CI) sig.[c]
Catkin	Autosomes and recombining Z	UB	1,754	0.0030 (0.0028 - 0.0031)	0.0135 (0.0130 - 0.0141)	0.2204 (0.2101 - 0.2311)	0.0030	0.0132	0.2232
		MB	674	**0.0032 (0.0030 - 0.0035) p = 0.012**	**0.0162 (0.0147 - 0.0187) p < 0.001**	**0.1951 (0.1769 - 0.2157) p < 0.001**	0.0032	0.0158	0.2017
		FB	732	0.0031 (0.0029 - 0.0033) p = 0.094	**0.0149 (0.0141 - 0.0158) p < 0.001**	0.2095 (0.1938 - 0.2256) p = 0.082	0.0031	0.0146	0.2128

[a] Unbiased (UB), male-biased (MB) and female-biased (FB) genes.

[b] Number of genes with both divergence and polymorphism data.

[c] *P* values based on 1,000 replicates permutation tests comparing male-biased and female-biased genes with unbiased genes. Significant *P* values (< 0.05) are shown in bold.

FIGURES

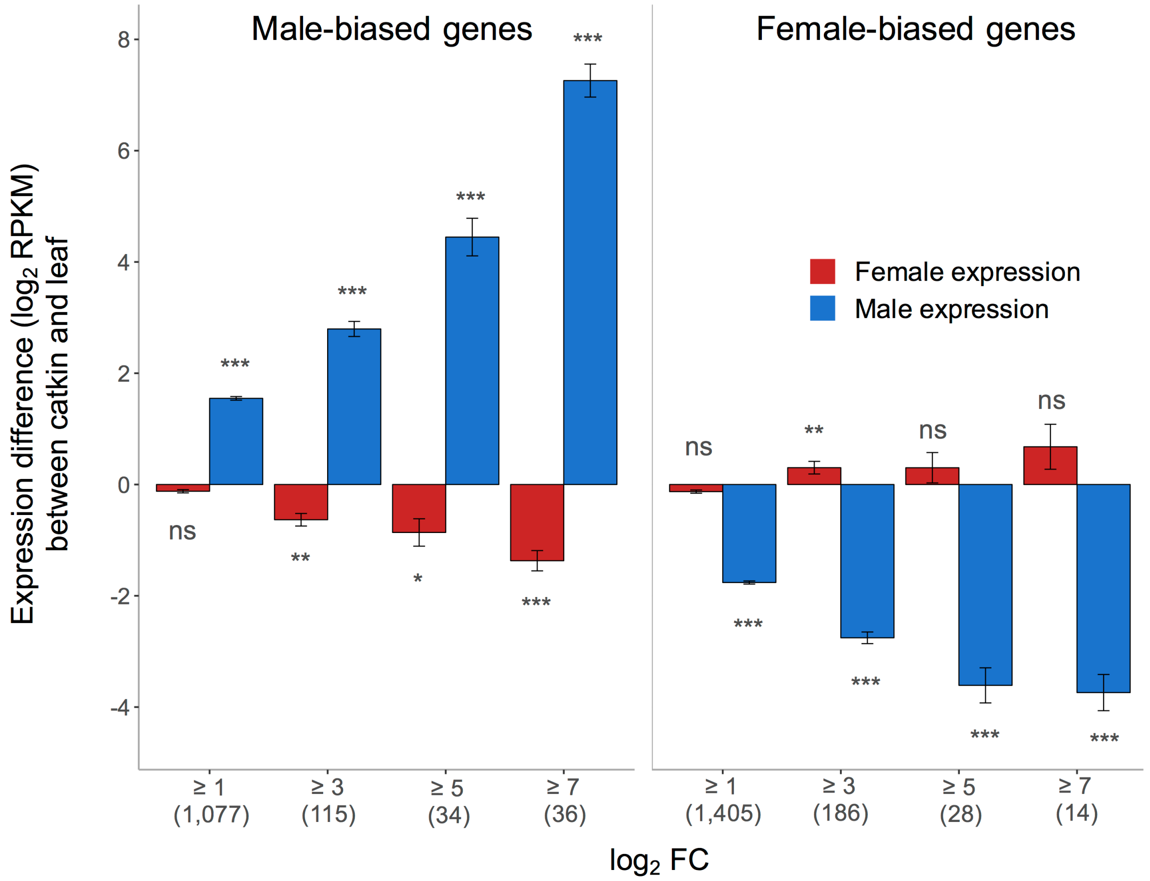

Figure S1: Expression differences between catkin and leaf samples (with standard errors of the mean) at different sex-bias fold change thresholds for male-biased and female-biased catkin genes. Male expression differences are shown in blue while female expression differences in red. Positive values indicate genes with higher expression in catkin than in leaf while negative values correspond to genes with higher expression in leaf compared to catkin. Significant differences in gene expression between the two tissues were determined through Wilcoxon rank sum tests (ns = non-significant, * = $P < 0.05$, ** = $P < 0.01$, *** = $P < 0.001$).

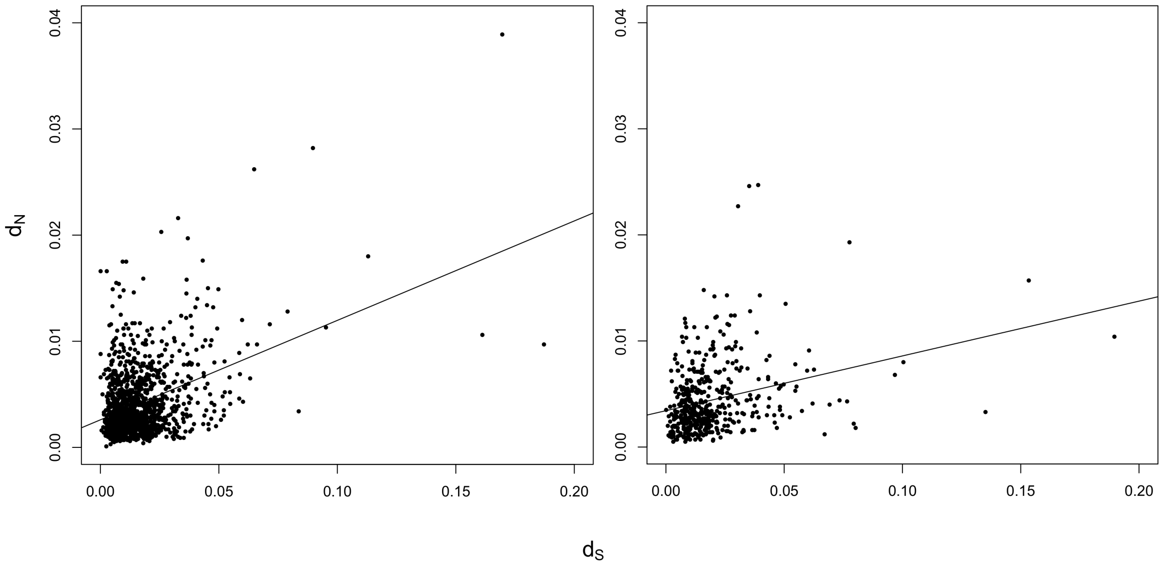

Figure S2: Relationship between the rate of synonymous (d_S) and nonsynonymous (d_N) substitutions for unbiased and male-biased genes.

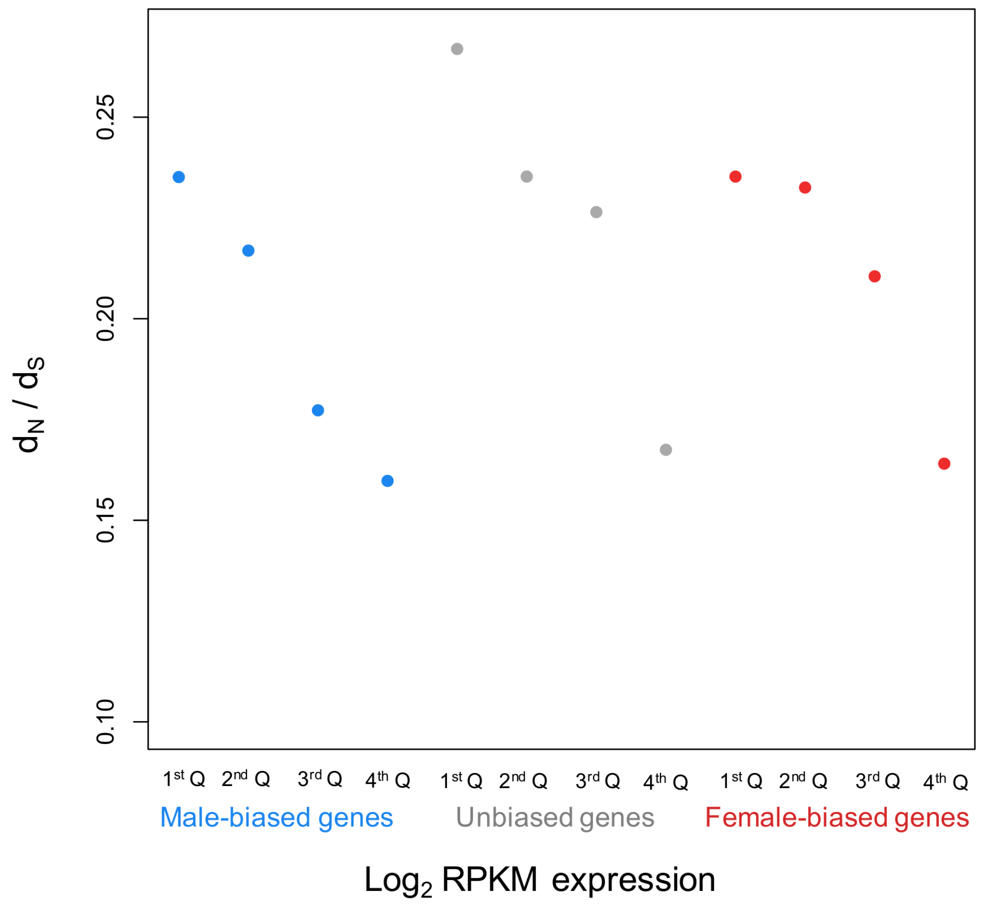

Figure S3: The ratio of nonsynonymous to synonymous nucleotide substitutions for male-biased, unbiased and female-biased genes. Both sex-biased and unbiased genes are divided into four equal quartiles of expression, where 1st quartile has the lowest expression and 4th quartile has the highest expression.

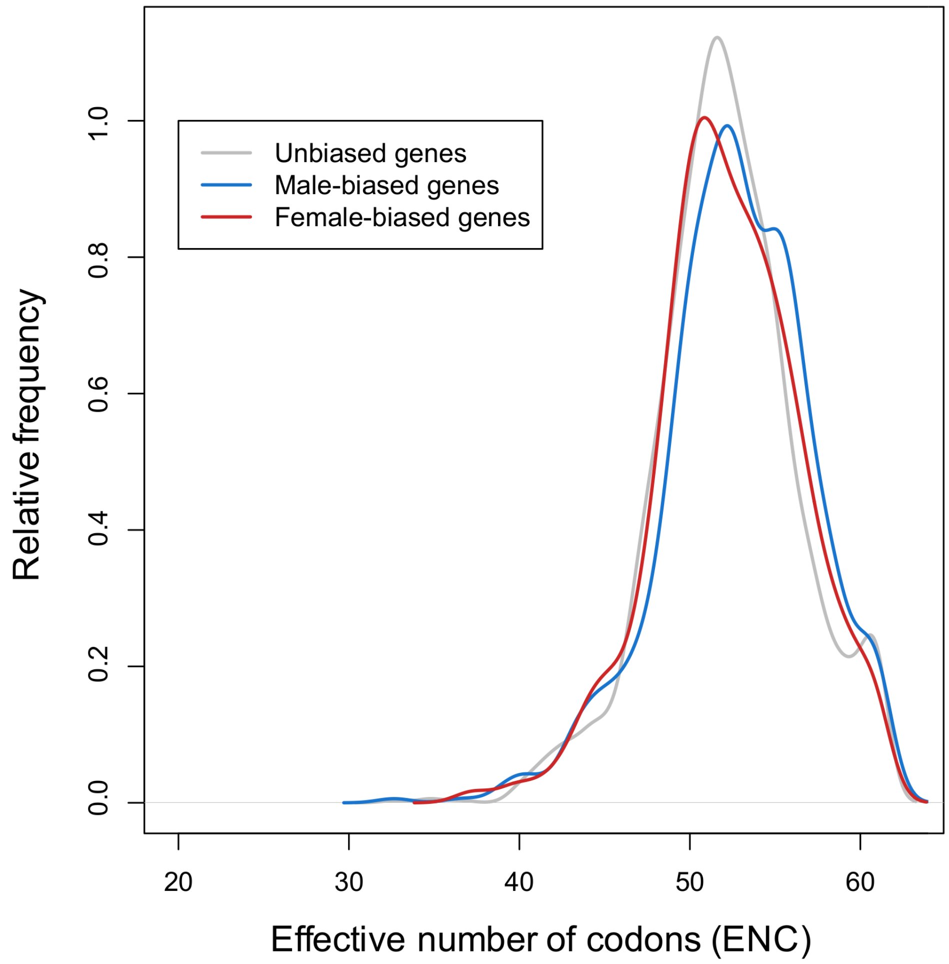

Figure S4: Density distribution of the effective number of codons (ENC) for unbiased (grey), male-biased (blue) and female-biased (red) genes. ENC is inversely related to the level of codon usage bias, ranging from 20 (extreme bias) to 61 (no bias).
